# Analyzing the Androgen Receptor Interactome in Prostate Cancer: Implications for Therapeutic Intervention

**DOI:** 10.3390/cells11060936

**Published:** 2022-03-09

**Authors:** Ujjwal R. Dahiya, Hannelore V. Heemers

**Affiliations:** Department of Cancer Biology, Lerner Research Institute, Cleveland Clinic, NB-40, 9500 Euclid Avenue, Cleveland, OH 44195, USA; dahiyau@ccf.org

**Keywords:** androgen deprivation therapy, hormonal therapy, transcription, coactivators, corepressors, proteomics

## Abstract

The androgen receptor (AR) is a member of the ligand-activated nuclear receptor family of transcription factors. AR’s transactivation activity is turned on by the binding of androgens, the male sex steroid hormones. AR is critical for the development and maintenance of the male phenotype but has been recognized to also play an important role in human diseases. Most notably, AR is a major driver of prostate cancer (CaP) progression, which remains the second leading cause of cancer deaths in American men. Androgen deprivation therapies (ADTs) that interfere with interactions between AR and its activating androgen ligands have been the mainstay for treatment of metastatic CaP. Although ADTs are effective and induce remissions, eventually they fail, while the growth of the majority of ADT-resistant CaPs remains under AR’s control. Alternative approaches to inhibit AR activity and bypass resistance to ADT are being sought, such as preventing the interaction between AR and its cofactors and coregulators that is needed to execute AR-dependent transcription. For such strategies to be efficient, the 3D conformation of AR complexes needs to be well-understood and AR-regulator interaction sites resolved. Here, we review current insights into these 3D structures and the protein interaction sites in AR transcriptional complexes. We focus on methods and technological approaches used to identify AR interactors and discuss challenges and limitations that need to be overcome for efficient therapeutic AR complex disruption.

## 1. AR Structure and Function 

**Figure 1:**
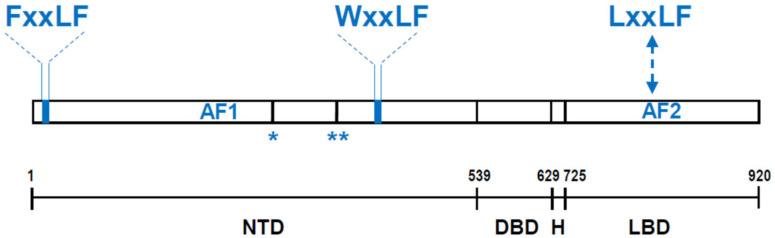
AR domain and motif organization. AR consist of an *N*-terminal domain (NTD) which contains a ligand-independent transcriptional function (AF) AF-1, a central DNA binding domain (DBD) and a C-terminal ligand-binding domain (LBD) which harbors the ligand-activated AF-2. DBD and LBD are connected by a hinge (H) region. FxxLF, WxxLF and LxxLF motifs represent short amino acid sequences relevant for AR domain interactions, AR transcriptional activity and AR-coregulator interaction, as discussed in the text. Numbers represent amino acids in AR protein, for which numbering is based on NM_000044.2. * and ** indicates a short 14 amino acid stretch and a proline rich region that has been used as bait in 2 hybrid screening or phage display assays in references He et al., 2004 and Blessing et al., 2015 respectively.

AR is a ligand-activated transcription factor that belongs to the nuclear receptor (NR) family [1,2]. Its modular structure resembles that of other NRs and consists of an *N*-terminal domain (NTD) which contains a ligand-independent transcriptional function (AF) AF-1, a central DNA binding domain (DBD) and a C-terminal ligand-binding domain (LBD) which harbors the ligand-activated AF-2. DBD and LBD are connected through a short hinge domain (Figure 1). 

**Figure 2:**
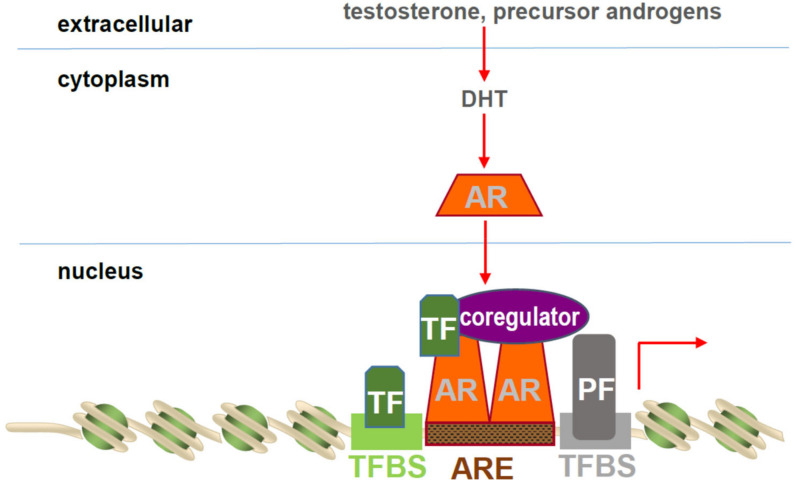
Basic mechanism of AR transcriptional activation. Testosterone or precursor androgens enter CaP cells and are converted into the most bioactive androgen dihydrotestosterone (DHT). DHT binding activates AR, which results in cytoplasmic to nuclear AR re-localization and AR dimerization. AR binds as a dimer to Androgen Response Elements (AREs) in AR target genes, where it associates with 3 classes of transcriptional regulators to mediate AR target gene transcription. These regulators consist of pioneer transcription factors (PFs), coregulators, and secondary transcription factors (TFs). TFBS, transcription factor binding site.

The manner in which AR is activated has been reviewed extensively in the past. To summarize, androgen binding to the AR LBD induces a conformational change, cytoplasmic to nuclear re-localization, and, following dimerization via intra- and intermolecular NTD-LBD interactions, binding of AR through its DBDs to Androgen Response Elements (AREs), the canonical AR binding site in target genes. ARE-bound AR then recruits transcriptional regulators to modulate the expression of target genes [3,4,5,6] (Figure 2).

**Figure 3:**
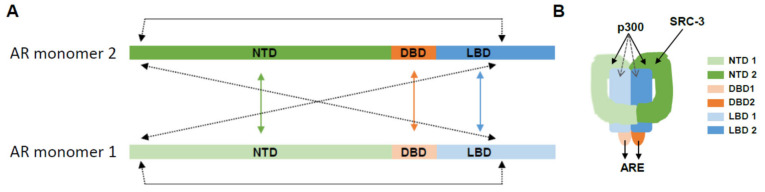
Contribution of AR domain interactions to AR dimerization, AR DNA binding and coregulator recruitment. (**A**) Current working model supports that AR dimerization occurs via intermolecular NTD-NTD, DBD-DBD and LBD-LBD interactions. Inter-and intramolecular NTD-LBD interactions further contribute to active AR conformation and dimerization. (**B**) Schematic representation of 3D AR domain interactions and interactions between AR domains and the AR-associated coregulators p300 and SRC-3. Black arrows, strongest interactions; dashed grey arrows, weak interactions. Data adapted from information provided in reference [11].

Until recently, the structure of full-length DNA-bound AR has remained elusive. Information on AR’s putative overall 3D conformation, and the contribution of AR’s domain interactions, homodimerization, and DNA-binding to the overall conformation of AR transcriptional complexes was derived from in vitro X-ray crystallography or computational modeling of individual LBD or DBD domains, either associated with small DNA fragments or small peptides from AR-associated proteins in the presence of a few ligands (e.g., [5,7]). The structure of the NTD, a highly disordered region which contributes considerably to AR action in ADT-resistant CaP [8,9,10], was completely unknown. In combination, these studies supported a working model, consistent with that for other NRs, in which ligand-induced AR dimerization is associated with repositioning of helix 12 in the LBD, which closes off the ligand binding pocket and forms a coactivator-binding surface [5]. Independent studies confirmed that NTD and DBD can also recruit transcriptional regulators [4]. A recent cryoEM study has revealed the first structure of full-length recombinant AR bound to in vitro ARE-containing DNA fragments [11]. In the latter study, the agonist-bound full-length ARE-DNA/AR structure showed the 2 LBDs and DBDs in the center of the dimer, with the 2 NTDs wrapping around the LBDs in close contact with each other. All AR domains contributed to the dimerization interface, and the NTDs also surrounded the LBDs to make both intra- and inter-molecular N/C interactions (Figure 3). The authors concluded that N/C AR interactions facilitate a tail-to-tail and head-to-head AR dimer formation. Two representative coregulators, SRC-3 and p300, interacted most strongly with the NTD at a ratio of 1 coregulator per AR dimer [11].

## 2. AR Action Drives CaP Progression

AR is widely expressed [12]. Because of its dependence on androgens, the main male sex steroids, it has been studied extensively in the context of the development and maintenance of the male phenotype [7,13]. None-the-less, AR activity demonstrates tissue-specificity and has been linked also to several conditions and diseases, such as alopecia [14] and spinal-bulbar muscular atrophy [15]. Most notably, AR has been recognized as a critical determinant of CaP progression. For 8 decades, AR has served as the major therapeutic target for the systemic treatment of metastatic CaP [16,17,18], which causes more than 30,000 cancer deaths in American men each year [19]. AR-targeting therapies, commonly referred to as androgen deprivation therapies (ADTs), were spurred by the landmark study by Huggins and Hodges, in which alleviation of CaP symptoms was reported after lowering circulating androgen levels derived from testes by surgical castration or high dose estrogens [20,21]. At that time, it was recognized that such therapy induced remissions but did not cure CaP. The recurrence of CaP during ADT is now known to result, at least in part, from diverse mechanisms of aberrant AR reactivation which cause CaP growth to remain driven by AR [9,18,22,23]. To overcome AR-mediated treatment resistance, subsequent and ongoing refinements to ADT have targeted the extra-testicular sources of androgen (precursor) production and maturation such as the adrenals and CaP cells [18]. As an alternative and sometimes complementary approach for androgen synthesis inhibition, so-called anti-androgens that prevent AR-androgen interaction are administered, with the potency of next generation AR inhibitors also increasing [18,22,24]. Resistance occurs to these novel generation AR inhibiting therapies. In a minority of cases, ADT-resistance can lead to development of neuroendocrine CaP (NEPC) that has become AR-indifferent, AR-negative or AR-independent [25,26]. The majority of recurrent cases, however, continue to depend on AR activity that is restored via numerous and ever expanding molecular mechanisms, which broadly encompass diverse ways of AR overexpression or amplification [23,27], mutations that render AR less sensitive to ADT [28], AR gene rearrangements or splicing events that result in loss of functional LBD [9,27] (and thus, a constitutively active AR), or expression of the related NR glucocorticoid receptor that in a subset of cases can take over part of AR’s function in CaP [29,30].

## 3. Novel Strategies to Target AR for CaP Treatment 

In view of AR’s central role in bypassing CaP’s response to the selective pressure of ADT, therapeutic strategies to inhibit AR’s activity other than impeding its ligand activation are being sought. Some of these approaches, which have not (yet) transitioned into the clinic, are directly targeting AR, such as AR degraders (ACS-J9 [31], AR PROTAC [32]) or are aimed at inactivating its ligand-independent transactivation function at the NTD (EPI compounds [33,34]). Alternative AR-inhibition approaches have been proposed that do not directly target AR (domains) or its stability, but instead prevent its association with proteins on which AR relies to execute its transcriptional program, which is how AR ultimately impacts CaP cell behavior [6,35,36]. 

Transactivation by ARE-bound AR is the result of the functional interplay between AR and proteins that belong to 3 major classes of transcriptional regulators [6]. The first class consists of pioneering transcription factors that directly bind condensed chromatin that is inaccessible to AR and then facilitate chromatin access so AR can form a complex to execute transcription of its target genes. The second group of proteins encompasses coregulators, which are generally non-DNA binding and harbor diverse functions (e.g., execute histone modifications, AR stabilization…) that assist AR in its transcriptional control over its target genes. Coregulators can either upregulate (act as a coactivator) or repress (act as a corepressor) transcription of AR target genes. The third class of AR transcriptional regulators, secondary transcription factors, further cooperate with AR to fine-tune ARE-driven transcription and either bind their own DNA recognition motif close to AREs or tether to DNA-bound AR (reviewed in [6], Figure 3). 

Hundreds of AR-associated proteins belonging to these 3 classes have been isolated to date [4,37,38]. The interest in preventing their physical interaction with AR transcriptional complexes for therapeutic development was fueled also by observations that several of these proteins were differentially expressed between benign prostate and treatment-naïve primary CaP and between primary CaP and treatment-resistant CaP [6,39]. At least for a subset of these proteins, deregulated expression can be linked with poor outcome, treatment resistance and CaP recurrence. The appeal of targeting the action of these AR interactors has increased because some have been suggested to contribute to context-dependent transcription of AR target genes [40,41], and may thus differentially impact CaP progression. Moreover, a subset (e.g., p300, BRD4) possesses enzymatic properties that are relevant to AR function and are druggable [42,43]. However, these AR interactors are generally not AR-specific but also modulate the activity of other transcription factors. Preventing their action may thus lead to off-target effects. Specific disruption of their interactions with AR using peptides, peptidomimetics or small molecules that target these sites or compete for interaction with the same AR-regulator binding sites is an appealing alternative [35,44]. The overall success of such strategies has been limited to date, at least in part by a lack of insights in the specific domains, sites and 3D conformations of interfaces by which interaction between AR and its transcriptional regulators occurs at the molecular level. A better understanding may lead to more effective and specific drugs.

Here, we review such knowledge, especially as it relates to technological approaches and methods used to identify AR interactors in CaP cells, and their AR interacting sites or domains. We discuss potential ways to alleviate challenges to such approaches and improve upon this knowledge, which is critical to achieve the proposed therapeutic strategy for alternative AR inhibition.

## 4. Screening for Novel AR Interactors

### 4.1. 2 Hybrid Assays

Since their conception in 1989 [45], 2 hybrid assays have been performed extensively to define novel protein–protein interactions. A significant portion of the AR protein interactome has been isolated using this technique. The basis for 2 hybrid systems is that protein–protein interactions between a “bait” protein (here: AR) and its prey(s) (here: AR interactors) result in reconstitution of the DNA binding and activating portion of a transcription factor (TF) (e.g., Gal4), which yields an active TF that controls expression of a gene encoding a critical nutrient or reporter gene that is easy to detect. Restoration of TF function is achieved by ectopic expression of a gene fusion that combines the DNA binding domain of the TF with the bait and a gene fusion that joins the TF’s activating domain with a prey protein. The prey is quite often expressed from a cDNA library, allowing for screening for novel bait interacting proteins. If bait and prey interact, cells either generate essential nutrients and survive, or induce a specific reporter signal or phenotype. These gene fusions are most commonly overexpressed in yeast, although bacterial, plant, insect, and mammalian cells can also be manipulated to facilitate 2 hybrid assays. The majority of 2 hybrid studies to isolate AR interactors have been done on yeast cells and have involved library screening approaches. The source for the latter cDNA libraries has been diverse and has consisted of normal human prostate cells and prostate cancer cells, but also, for instance, genital tubercle tissues of male mice, human fetal brain, human testis and human male monocytes [46,47,48,49,50,51,52,53,54,55,56]. 

The advantages of the 2 hybrid assays are many, including its cost effectiveness, scalability, potential for automation and its relatively low technological requirements which can be easily implemented in most laboratories. Yet, because the assay relies on overexpression of protein (fragments) in the cell nucleus that may either not be expressed endogenously or not expressed at the same level or in the same cell compartment in human prostate cancer cells, it is prone to false-positive results. The absence of post-translational modifications in yeast that do occur in mammalian cells adds to this problem, as does the observation that traditional yeast 2 hybrid assays do not perform optimally when the bait protein possesses intrinsic transactivation activity, such as the AR NTD. Some of these issues can be overcome, for instance, by co-expression of a kinase in yeast cells to ensure the presence of relevant phosphorylation marks [57] or the use of a split-ubiquitin system which facilitates isolation of interactors for integral membrane proteins [58]. Similarly, modifications to the traditional 2 hybrid assays have been developed, such a repressed trans-activator system, which has allowed identification of several novel AR NTD interaction proteins (e.g., DDC, TAF1 and GAK) [54,56,59]. Internal controls, e.g., site-directed mutagenesis in the putative interaction sites, can be incorporated in the 2-hybrid assay. Moreover, independent technical validation of 2 hybrid assay results have routinely included GST pull-down or co-IP studies, while functional validation of the putative AR interactor on AR transcriptional function typically involves AR-driven promoter-reporter assays, qRT-PCR on endogenously expressed AR target genes or gene-wide transcriptomics analyses for AR/androgen-dependent events. 

Structural and/or conformational information on the AR transcriptional complex that can be derived from 2 hybrid assays, however, appears very limited. The focus on different AR domains, regions, and even short AR amino acid sequences that are conserved in AR among species (overview in Table 1) in an in vitro setting does limit considering the full implications on AR complex formation. Nonetheless, despite these limitations, several AR-associated coregulators and transcription factors, including, for instance, ARA-70 [60] that are now well-recognized as critical determinants of AR function have been derived from 2 hybrid assays. Table 1 provides an overview of representative AR domains and motifs used as bait, source of libraries for prey, and validation approaches for representative examples of AR interactors isolated via 2 hybrid assays. 

### 4.2. Phage Display Assays

The phage display assay represents another method that is commonly used to screen for novel protein-protein interactions [61,62], and has also led to the isolation of AR interactors. The principle of this assay is that an immobilized bait protein (here: AR (domain)) is exposed to bacteriophages that have been engineered to express prey proteins as inserts in their phage coat proteins. The phage displays the prey protein on its outside, which allows the interaction with the bait. Phages expressing prey proteins that bind the bait at high affinity are retrieved and used to infect bacteria to produce more phage. Multiple rounds of selection and amplification are performed to enrich for higher affinity binding phages compared to the previous round, until the DNA sequence for the prey from the highest affinity binding phage is isolated, sequenced and the interacting protein is identified. Several phage systems are available, such as T7 and filamentous phages, which have both been applied successfully to identify novel AR interactors. These phage systems differ in the size of the proteins they can accommodate (with filamentous phages facilitating the expression of larger proteins) and the type of protein they can express on their surface (with T7 phages facilitating expression of proteins that cannot be translocated across the bacterial inner membrane before they are assembled into the filamentous phage) [63,64]. 

As bait protein, full length AR has been used, as well as AR domains (e.g., LBD) and short AR motifs (e.g., proline-rich region in the AR NTD (see Figure 1)). Prey fusions can be expressed from a cDNA library from prostate (cancer) or other cells or can be derived from a library of peptides. While both approaches have yielded novel AR interactors (e.g., FHL2 from a cDNA library strategy [65]), the peptide library-based screens especially gained a lot of traction. Interest in the latter was fueled by a previous body of work linking short peptide sequences in coregulators or in AR with NR interaction or AR activation, respectively (marked in Figure 1). Indeed, an LxxLL motif present in several coregulators preferentially mediates their interaction with the LBD and AF2 groove in other NRs [66,67,68], suggesting that such motifs could be exploited to isolate pivotal AR interactors. In addition, the well-recognized NTD-LBD terminal interaction that is important for AR’s transcriptional activity is mediated at least in part by a FxxLF motif in the AR NTD [69]. Yet another AR NTD motif, WxxLF, has also been shown as critical to execute AR’s transcriptional activity (Figure 1) [69,70]. In view of the importance of these peptide sequences for AR function, several studies have screened libraries of hundreds of millions of short peptides that included extensive variations of the LxxLL, FxxLF and WxxLF sequences [71,72,73,74,75]. In addition to deriving novel AR-interacting motifs, these efforts revealed that the majority of peptides that bind AR with high affinity are more aromatic than the LxxLL motif that strongly binds other NRs—the motif that consistently bound AR more effectively across multiple studies was FxxLF [71,73,74]. Even though the prey peptides used in these screens could be short, around 12 amino acids, via in silico analyses the presence of high affinity binding peptides was verified in endogenously expressed full length proteins. For instance, BUD31 and SH3YL1 were identified as novel coregulators using such approaches [74,75,76]. Assay controls routinely include the modification of peptides present in the screen, which allows for enrichment of highest affinity binders, or site-directed mutagenesis, e.g., mutation/deletion of proline residues in the above-mentioned AR bait region. Technical validation is achieved in a variety of manners including mammalian 2 hybrid assays, FRET, GST pulldown, and Co-IP studies. The impact of the interaction on AR function is validated by overexpression, silencing or site-directed mutagenesis of the identified proteins or peptides on AR target gene expression and AR-driven promoter-reporter genes.

Phage display assays do suffer for some of the limitations mentioned above for 2 hybrid assays; they also rely on (over)expression of protein domains or peptides that may not fully represent endogenous post-translational modifications and localization. Limitation in terms of size of library or type of protein that can be expressed is mentioned already above. On the other hand, phage displays tend to be easy to perform, relatively cheap, adaptable and amenable to high throughput approaches. They are able to select peptides and proteins with high affinity and specificity that may be developed further and moved forward as preclinical AR-inhibitory therapeutics. As proof-of-principle, an FxxLF-containing AR-interacting peptide functioned as peptide antagonist of AR-dependent transcription [73]. Phage display allows for more detailed characterization of newly isolated AR interactions than 2 hybrid assays as the peptide binding affinity for observed interactions can be calculated easily. For instance, surface plasmon resonance (SPR) or FRET assays can inform on the equilibrium constants between AR and the peptides it interacts with. 

From a conformational and structural perspective, important novel information ensued from phage display assay findings. Apart from fine-tuning the protein/peptide sequences that most stringently interact with AR, deeper insights in AR structure-function relationships and its allosteric regulation have been obtained. First, AR-binding peptides that reflect variations on the LxxLL, FxxLF, and WxxLF motif theme have been combined with the AR LBD in X-ray crystallography studies [71]. These efforts revealed binding of all peptide subtypes to the same overall binding surface, the AF-2 groove, with changes in the surface occurring based on specific peptide-AR interactions [71]. Such an induced fit model is consistent with allosteric regulation of AR function [77]. Additional studies took into consideration the effect of AR agonists, antagonists and SARMs on AR-interactor binding identified in phage display assays. Phage display in which preys were expressed from a cDNA library and full-length AR used as bait recognized 8 subclasses of AR binding patterns based on differential presentation of protein interaction surfaces on the AR that were each impacted variably by different AR ligands [78]. Such studies illustrate the potential of phage display to help define the pharmacological properties of bound ligands, and to isolate novel AR inhibitors [36,73]. They again support context-dependent activation and conformation of AR that is dictated by specific protein and ligand interactions [77]. Finally, from a therapeutic perspective, overexpression of peptides that bind AR and are derived from phage display studies, e.g., the AR-interacting motif in BUD31, inhibited transcriptional activity of AR and CaP cell viability and proliferation [74]. The latter findings indicate that results from phage display studies can provide a first glimpse into the therapeutic potential of AR-regulator interactions and serve as proof of principle that disrupting AR transcriptional complex may be a viable therapeutic strategy in late-stage CaP. An important caveat remains that these preclinical findings have not yet been carried forward in clinically relevant models or clinical trials. The extent to which point mutations in AR that occur under ADT impact these interactions and their therapeutic potential is also not yet clear, as some studies reported alterations in binding affinity between wild type AR, present in treatment-naïve CaP, and mutated versions of AR (e.g., AR T877A) that are found at increasing frequency in CRPC [75].

Other approaches that have the potential to screen for novel AR interactors have, to our knowledge, not yet been used (extensively) for this purpose and are mentioned here for completeness. For instance, protein arrays have a similar set up as DNA (oligo) arrays but involve spotting proteins instead of DNA on a slide to screen for interactors from cell or tissue lysates. The commercial Marconi peptide array uses NR-coregulator derived peptides to screen for NR interactors and showed interaction between an LxxLL motif in the coregulator TRIM24 and the AR LBD [79]. It should be noted also that AR interactors have been derived also from reversed order 2 hybrid and phage display experiments in which AR was isolated a prey for baits with a coregulator role, e.g., KLF8 [80].

## 5. Characterizing the Composition of AR Transcriptional Complexes

The assays discussed above represent screens for novel AR interactors that were performed in non-CaP model systems. Alternative strategies to identify AR-associated cofactors and coregulators have focused on better defining the composition of the endogenous AR transcriptional complexes in CaP cells. A number of approaches that vary in bait protein purification steps, stabilization and/or labelling of protein-protein interactions and are coupled to mass spectrometry (MS)-based identification of AR interactors have been applied to this end.

### 5.1. Affinity Purification Mass Spectrometry

In this first approach, various antibody- or tag-based methods are used to affinity-purify (AP) the bait protein (here: AR) and its interactors from CaP cells or tissues. The purified bait and its associated complex are then washed extensively to eliminate non-specific interactions and subjected to a MS approach for characterization The whole protein eluate can be subjected to MS, which allows for a more unbiased characterization of AR associated proteins. Alternatively, a more selective approach, which narrows down the search for interactors based on size and concentrates samples, involves running the pulled-down proteins on an SDS-PAGE gel, excising the gel fragment of interest and enzymatic digestion of this fragment prior to MS data acquisition. Further refinements to AP-MS are possible. For instance, IPs can be done from a specific cell compartment, such as the nucleus to enrich for proteins with a transcriptionally relevant function. Enrichment for activators or repressors of AR action can be achieved by first treating cells with AR agonists or antagonists.

To better understand AR complex composition, several studies used GST-tagged AR that was immobilized on beads or columns as bait and isolated interactors from CaP cell lysates were identified via MS analysis. A study that screened for interactors of GST-labelled AR *N*- and C-terminal domains purified Ku70 and Ku80 as AR coactivators [81]. In others, AP-MS assays used AR-targeting antibodies to pull down AR at its endogenous expression levels and its regular cellular location. Such studies have been instrumental in clarifying the composition of endogenous AR complexes and have revealed novel AR interactors and/or confirmed interactors derived from above mentioned screening efforts (e.g., USP7, ref. [82]). An advantage of such strategies is that they could be applied on cell line models as well as on CaP tissues, either from animal models or patient specimens. Provided that enough starting material of sufficient quality is available, AP-MS could detect also post-translational modifications on AR (interactors), which are known to be relevant e.g., for protein-protein interactions or activity status of AR. As an example, a modification such as phosphorylation can be deduced from MS data analysis from assays starting from bait-targeting antibodies or following IPs with phospho-Ser/Thr/Tyr-motif-specific antibodies. The latter approach may be amenable to analyze clinical specimens, as similar motif specific-antibodies and phospho-proteomics have been applied to deduce kinome pathway activity in treatment-resistant CaP [83,84]. The overall procedure can be adjusted also to include a phospho-enrichment step, using for instance TiO beads following AP and prior to MS, which allows to enrich further for phosphorylated proteins/peptides. The MS assay analysis following AP has been done in various ways that can be based on size, charge, or predictions or a combination thereof. Validation of results from AP-MS approaches, which are prone to yield false-positives, generally include co-immunoprecipitation (coIP), immunofluorescence or proximity ligation assays to verify protein-protein interactions and colocalization. ChIP and gene expression studies are done to verify recruitment to AREs and AR target gene levels.

An important limitation to these assays, however, is that they rely on the availability of a specific antibody that is suitable for efficient immunoprecipitation (IP). This problem can be overcome by using tagged versions of the bait protein, in which for instance a FLAG, His, GFP, Myc or HA tag is fused in frame that can be readily IP’ed with a commercial antibody. The tagged bait is then overexpressed in cells prior to IP with a tag-targeting antibody, more effectively IP’ed and its interactome defined. Since this method does lead to artificially high cellular levels of proteins, it may skew the ratios with interactors, protein interfaces and access to epitopes. This may be overcome by careful titrating overexpressed proteins or combining their ectopic expression with depletion of the endogenously expressed bait protein. The addition of a tag and the site to which it is added (*N*- or C-terminal) may influence the normal bait expression levels of folding patterns and thereby its function and interactome. None-the-less, using tagged versions of AR in AP-MS has expanded the AR interactome, for instance ChREBP [85] has been isolated and validated as AR coregulator in this manner. 

### 5.2. Rapid Immunoprecipitation Mass Spectrometry of Endogenous Proteins

A general drawback to the approaches above is that they may not efficiently detect protein-protein interactions that are labile or transient. A recent adaptation to overcome such limitations has involved the inclusion of a crosslinking agent that preserves these interactions. In Rapid Immunoprecipitation Mass spectrometry of Endogenous proteins (RIME), formaldehyde is used to crosslink endogenous protein-protein interactions [86,87]. Formaldehyde is already used in ChIP(-Seq) assays to crosslink DNA-protein interactions; these techniques are complementary, and their combination has proven useful to identify and characterize coregulators and cofactors of DNA-bound transcription factors. This assay can be done on smaller amounts of starting material, which makes it amenable also to tissue samples. Antibodies targeting the bait are immobilized on beads, to which sonicated nuclear lysates from crosslinked cells are added. Beads are washed extensively, eluated and the eluate subjected to enzymatic digestion prior to MS analyses. Several groups have applied RIME to CaP cells to gain better insights into AR function. For instance, comparing protein interactomes between isogenic CaP cells that express either full length AR or the AR variant Arv567es using RIME confirmed many known AR coregulators such as FoxA1, p300 and identified GRHL2 as a novel AR-associated coregulator [88]. Another study performed RIME on the chromatin remodeler CHD1 and found significant overlap between the interactomes for CHD1 and AR [89].

### 5.3. Biotin-Based Proximity Ligation Assay

Another way to overcome the problems with isolating transient or labile protein-protein interactions and to focus on proteins in the immediate vicinity of the bait is evidenced by biotin-based proximity labeling assays. In such assays, a promiscuous biotin-labeling enzyme is targeted by genetic fusion to the bait protein and to a subcellular compartment. The enzymes used in these assays are constantly being refined. Currently, TurboID has substantially higher activity than the previously described BioID [90]. A recent version, Split-TurboID, consists of two inactive fragments of TurboID that can be reconstituted through protein-protein interactions or organelle-organelle interactions, which fosters even greater targeting specificity [91]. The addition of a small molecule substrate such as biotin initiates covalent tagging of endogenous proteins within a 10 nm radius of the enzyme [90]. Biotinylated proteins can be retrieved using streptavidin-coated beads and defined using MS on the beads to identify proteins proximal to the fused AR that were biotin-tagged by the Turbo-ID-fusion protein. Although this relatively novel technique has not yet extensively used in CaP cells, it has recently identified KLF4 as AR interactor [92].

In combination, these MS-based characterization efforts on AR transcriptional complexes have considerably expanded the spectrum of its coregulators and cofactors. Alternatives are possible, for instance (MS) analyses of complexes bound to ARE-containing regions [93,94]. A limitation of the approaches above is the potential for aspecific interactions or false positives to be detected by MS, emphasizing the need for stringent analysis and experimental validation of MS data [95]. Along the same lines, despite isolation of multi protein AR complexes, information on their conformation and 3D structures, needed for the proposed therapeutic strategy, has been difficult to ascertain from such studies. Ongoing evolution in MS-based assays, e.g., crosslinking (XL)-MS, that allows to assess the proximity of amino acids and to infer protein folding and complex topology, may help alleviate these constraints and provide important insights in protein interfaces [96]. 

## 6. Opportunities, Challenges and Limitations for Effective Therapeutic AR Complex Disruption

AR’s role in CaP biology is tightly regulated by its interaction with other cellular proteins which affect its binding to ligand, nuclear translocation, folding and transcriptional activity. Over the past few decades, hundreds of AR-interacting proteins have been identified using different unbiased approaches [4,37]. Together, the approaches described above have each contributed to the identification of the current spectrum of AR-associated coregulators and cofactors. Several of these regulators were identified as AR interactors by at least 2 independent screening and/or characterization assays described above. Representative examples of these, their cellular localization and functions are included in Table 2 [56,82,88,92,97,98]. AR-interacting proteins display remarkable functionally diversity and are involved in various cellular processes or signal transduction pathways [4,37]. Their combined role in mounting an AR transcriptional output, along with the isolation of the AR cistrome [99], is starting to uncover the breath of the biology under control of AR and the context-dependency of AR action. At the same time, questions arise as to how to leverage this information to develop novel CaP therapeutics that target transcription factor function of AR. 

That disrupting AR complexes may have therapeutic potential when AR-driven CaP has lost responsiveness to ADT is supported by a multitude of studies. In these reports, expression of AR-associated coregulators or cofactors is silenced by siRNA, shRNA or CRISPR approaches, or dominant-negative or catalytic-dead versions of diverse coregulators are overexpressed. Moreover, coregulator-derived decoy proteins or peptides that compete with coregulators for AR binding have shown promise in model systems after short term treatments [44,73]. Peptidomimetics designed to inhibit AR’s interaction with NR box proteins through their LxxLL motif reduced ligand-stimulated nuclear localization of AR as well as CaP cell proliferation [35]. Yet none of these strategies have transitioned into clinic. 

The structure-function basis for the majority of these AR-regulator interactions is yet to be defined, which will likely be critical to explore their full potential for therapeutic intervention [100]. Effective and specific targeting the functional interplay between AR and its associate transcriptional regulators will likely require detailed insights in the molecular basis for their physical interactions, including not only the precise and selective interaction sites but also the 3D conformation of these interfaces and that of the larger AR complex and multimer. Using the approaches described above, some studies have been able to retrieve some useful information and have for instance fine-tuned the specific amino acid motifs by which coregulators can preferentially interact with AR [71,74,101] or have defined crystal structure of cofactor-derived peptides associating with AR regions [71,74]. Others have delineated the effects of AR agonists, antagonists or SARMs on their association, leading to grouping of coregulators based on interaction patterns after ligand treatments [78]. However, these assays mostly involved interaction of short peptides with isolated AR domains in an in vitro setting, so their results may not necessarily be representative of the molecular interactions in endogeneously expressed full-length proteins. 

The first cryoEM study on full length DNA-bound AR has recently been published [11]. As discussed above, this work has provided new insights in the manner in which AR engages in intra- and intermolecular dimerization. Importantly, this study also provided new insights in domains (NTD) by which pivotal AR coactivators SRC-3 and p300 preferentially interact with AR, and the stochiometry by which they associated with AR (1 coactivator each per AR dimer). Insights from such assays on more and other coregulators may provide new information on (conformation of) interfaces between AR and its regulators and the ratios in which different components make up the AR transcriptional complex. While the above-mentioned study was done in vitro, in vivo cryoEM assays on endogenous protein complexes have been reported [102], which may provide additional information on how to extrapolate findings to the endogenous setting. Alternatively, or complementarily, cross-linking mass spectrometry (XL-MS) has emerged as another method to provide lower resolution modeling of protein (complex) conformations and protein-protein interactions [96]. This technique is applicable to in vivo protein complexes [96,103], and has already validated conformational changes for PPAR-gamma [104], suggesting it may be amenable to study other NRs such as AR. In combination with computational modelling and bioinformatics tools that are continuously evolving and being refined, new insights in AR complex formation may be derived. 

In anticipation of the renewed insights in AR complex composition, conformation and stoichiometry that the above-mentioned technological advances and others that may emerge can deliver, it is important to consider a few questions will need to be addressed and prioritized to deliver the most useful information that can be move forward clinically. For instance, to which of the hundreds of AR-associated transcriptional regulators should these efforts be directed to ensure clinical applicability? It seems reasonable that an AR interactor pursued for therapeutic intervention contributes to AR activity that drives ADT-recurrent CaP progression. Its AR-dependent activity or expression may increase also during CaP progression. Ideally, its function would not be impacted by CaP genomic heterogeneity [105]. On the other hand, an interactor may become subject to genomic alterations in a subset of CaP cases which may render it more amenable for targeting and may create a therapeutic window that facilitates precision medicine interventions. Ideally, its AR interaction site(s) are AR-specific and also CaP- or prostate-specific so that significant off target effects and toxicity do not occur.

With respect to the proposed therapeutic targeting, several other questions remain. From a molecular perspective, as protein–protein interactions in transcriptional complexes display a tiered hierarchy [106], what level of complex resolution is necessary and at which tier are disruptions best induced to inhibit AR function? How to best consider and capture dynamic protein-protein interactions and the kinetics of AR complex formation? AR’s interaction with ligands, associated proteins and DNA recognition motif all induces discrete conformation changes in distinct AR regions [71,77,78]. Can and should the different context-dependent AR conformations that contribute to AR’s overall activity be resolved and targeted for therapeutic targeting? How should the impact of different AR expression levels, somatic mutations and variants, which may impact dimerization, folding or access to interaction sites, be taken into account? Should these efforts be directed to DNA-bound AR only?

Considering and prioritizing these relevant issues will be important to ultimately employ the anticipated novel molecular insights in AR complex formation in an evidence–based manner for therapeutic intervention in treatment-resistant CaP.

## Figures and Tables

**Table 1:** Representative sample of AR interactors identified via 2 hybrid screening. For each AR interactor, the table shows the AR fragment or domain used as bait, the AR domain the prey preferentially interacts with, the cDNA library used in the assay, the assays used to validate interaction with AR and impact on AR function and the relevant literature report. Please note that numbering of amino acids (aa) reflects the numbers that were included in the original research reports.

AR Interactor	Bait	AR Interacting Domain	Library	Validation Assays	Reference
**ARA267-α**	aa 595-918	DBD + LBD	human brain	in vitro coIP, transient tranfection	[48]
**CHIP**	aa 220-270	NTD	human testis	in vitro coIP, mammalian 2 hybrid, immunocytochemistry, transient transfection	[47]
**DDC**	aa 1-559, aa 233-559, aa 1-646	NTD, LBD	LNCaP cells	in vitro and in vivo coIP, transient transfection	[54]
**FOXG1**	aa 325-919	DBD + hinge + LBD	human fetal brain	1 and 2 hybrid, in vitro and in vivo coIP, transient transfection	[51]
**HBO1**	aa 505-919	DBD + LBD	human prostate	in vitro and in vitro coIP, transient transfection	[49]
**p68**	DBD + LBD		human prostate	in vivo coIP, immunofluorescence, ChIP, transient transfection	[52]
**PRMT10**	aa 1-98	NTD	universal human	in vitro and in vivo coIP, mammalian 2 hybrid, AR-dependent cell proliferation	[53]
**RanBPM**	aa 1-232	NTD, DBD	human prostate	in vitro and in vivo coIP, transient transfection	[46]
**RWDD1**	aa 555-920	LBD	genital tubercle of male mice	in vitro and in vivo coIP, transient transfection	[50]
**Sertad1**	LBD	LBD	male peripheral blood monocytes	in vitro and in vivo CoIP, immunocytochemistry	[55]
**TAF1**	aa 1-559, aa 233-559, aa 1-646	NTD	LNCaP cells	in vitro and in vivo coIP, ChIP, transient transfection	[56]

**Table 2:** Representative examples of AR interactors identified via 2 independent screening or characterization approaches. For each AR interactor, the table shows the first and the second method by which the protein was isolated, the reference for both papers reporting the interactor as associated with AR, the cellular localization of the interactor and its function. Y2H, yeast 2 hybrid; PLA, proximity-ligation assay; RIME, Rapid Immunoprecipitation Mass spectrometry of Endogenous proteins; AP-MS, affinity purification mass spectrometry.

AR Interactor	Method 1	Ref 1	Method 2	Ref 2	Cellular Location	Function
TAF1	Y2H	[56]	PLA	[92]	nucleus	transcriptional regulator
NCOR1	PLA	[92]	RIME	[88]	nucleus	coregulator
USP7	AP-MS	[82]	PLA	[92]	cytoplasm, nucleus	ubiquitinyl hydrolase
SRC1	Y2H	[97]	PLA	[92]	cell junction, cell membrane, mitochondrion, perinuclear region	coregulator
RBM10	PLA	[92]	RIME	[98]	nucleus	RNA binding
SSBP2	PLA	[92]	RIME	[88]	cytoplasm, nucleus	splicing
SMARCE1	RIME	[98]	PLA	[92]	nucleus	chromatin remodeller

## Data Availability

Not applicable.

## References

[1] Mangelsdorf D.J., Thummel C., Beato M., Herrlich P., Schütz G., Umesono K., Blumberg B., Kastner P., Mark M., Chambon P. (1995). The nuclear receptor superfamily: The second decade. Cell.

[2] McKenna N.J., Cooney A.J., DeMayo F.J., Downes M., Glass C.K., Lanz R.B., Lazar M.A., Mangelsdorf D.J., Moore D.D., Qin J. (2009). Minireview: Evolution of NURSA, the Nuclear Receptor Signaling Atlas. Mol. Endocrinol..

[3] Heinlein C.A., Chang C. (2002). Androgen receptor (AR) coregulators: An overview. Endocrinol. Rev..

[4] Heemers H.V., Tindall D.J. (2007). Androgen Receptor (AR) Coregulators: A Diversity of Functions Converging on and Regulating the AR Transcriptional Complex. Endocrinol. Rev..

[5] Helsen C., Claessens F. (2014). Looking at nuclear receptors from a new angle. Mol. Cell. Endocrinol..

[6] Kumari S., Senapati D., Heemers H.V. (2017). Rationale for the development of alternative forms of androgen deprivation therapy. Endocr. Relat. Cancer.

[7] McEwan I.J., Brinkmann A.O., Feingold K.R., Anawalt B., Boyce A., Chrousos G., de Herder W.W., Dhatariya K., Dungan K., New M., Sahay R., Levy M. (2000). Androgen Physiology: Receptor and Metabolic Disorders. Endotext.

[8] Quayle S.N., Mawji N.R., Wang J., Sadar M.D. (2007). Androgen receptor decoy molecules block the growth of prostate cancer. Proc. Natl. Acad. Sci. USA.

[9] Ho Y., Dehm S.M. (2017). Androgen Receptor Rearrangement and Splicing Variants in Resistance to Endocrine Therapies in Prostate Cancer. Endocrinology.

[10] McEwan I.J. (2012). Intrinsic disorder in the androgen receptor: Identification, characterisation and drugability. Mol. BioSyst..

[11] Yu X., Yi P., Hamilton R.A., Shen H., Chen M., Foulds C.E., Mancini M.A., Ludtke S.J., Wang Z., O’Malley B.W. (2020). Structural Insights of Transcriptionally Active, Full-Length Androgen Receptor Coactivator Complexes. Mol. Cell.

[12] Davison S.L., Bell R. (2006). Androgen physiology. Semin. Reprod. Med..

[13] Marker P.C., Donjacour A.A., Dahiya R., Cunha G.R. (2003). Hormonal, cellular, and molecular control of prostatic development. Dev. Biol..

[14] Ceruti J.M., Leirós G.J., Balañá M.E. (2018). Androgens and androgen receptor action in skin and hair follicles. Mol. Cell. Endocrinol..

[15] Querin G., Bede P., Marchand-Pauvert V., Pradat P.-F. (2018). Biomarkers of Spinal and Bulbar Muscle Atrophy (SBMA): A Comprehensive Review. Front. Neurol..

[16] Denmeade S.R., Isaacs J.T. (2002). A history of prostate cancer treatment. Nat. Rev. Cancer.

[17] Schmidt L.J., Tindall D.J. (2013). Androgen receptor: Past, present and future. Curr. Drug Targets.

[18] Dai C., Heemers H., Sharifi N. (2017). Androgen Signaling in Prostate Cancer. Cold Spring Harb. Perspect. Med..

[19] Siegel R.L., Miller K.D., Fuchs H.E., Jemal A. (2022). Cancer statistics, 2022. CA Cancer J. Clin..

[20] Huggins C.S. (1941). Studies on prostatic cancer: 2. The effects of castration on advanced carcinoma of the prostate gland. Arch. Surg..

[21] Huggins C., Hodges C.V. (1941). Studies on Prostatic Cancer. I. The Effect of Castration, of Estrogen and of Androgen Injection on Serum Phosphatases in Metastatic Carcinoma of the Prostate. Cancer Res..

[22] Watson P.A., Arora V.K., Sawyers C.L. (2015). Emerging mechanisms of resistance to androgen receptor inhibitors in prostate cancer. Nat. Rev. Cancer.

[23] Viswanathan S.R., Ha G., Hoff A.M., Wala J.A., Carrot-Zhang J., Whelan C.W., Haradhvala N.J., Freeman S.S., Reed S.C., Rhoades J. (2018). Structural Alterations Driving Castration-Resistant Prostate Cancer Revealed by Linked-Read Genome Sequencing. Cell.

[24] Tran C., Ouk S., Clegg N.J., Chen Y., Watson P.A., Arora V., Wongvipat J., Smith-Jones P.M., Yoo D., Kwon A. (2009). Development of a Second-Generation Antiandrogen for Treatment of Advanced Prostate Cancer. Science.

[25] Davies A.H., Beltran H., Zoubeidi A. (2018). Cellular plasticity and the neuroendocrine phenotype in prostate cancer. Nat. Rev. Urol..

[26] Beltran H., Demichelis F. (2021). Therapy considerations in neuroendocrine prostate cancer: What next?. Endocrinol. Relat. Cancer.

[27] Henzler C., Li Y., Yang R., McBride T., Ho Y., Sprenger C., Liu G., Coleman I., Lakely B., Li R. (2016). Truncation and constitutive activation of the androgen receptor by diverse genomic rearrangements in prostate cancer. Nat. Commun..

[28] Nelson W.G., Yegnasubramanian S. (2013). Resistance Emerges to Second-Generation Antiandrogens in Prostate Cancer. Cancer Discov..

[29] Arora V.K., Schenkein E., Murali R., Subudhi S.K., Wongvipat J., Balbas M.D., Shah N., Cai L., Efstathiou E., Logothetis C. (2013). Glucocorticoid Receptor Confers Resistance to Antiandrogens by Bypassing Androgen Receptor Blockade. Cell.

[30] Li J., Alyamani M., Zhang A., Chang K.-H., Berk M., Li Z., Zhu Z., Petro M., Magi-Galluzzi C., Taplin M.-E. (2017). Aberrant corticosteroid metabolism in tumor cells enables GR takeover in enzalutamide resistant prostate cancer. eLife.

[31] Yang Z., Chang Y.-J., Yu I.-C., Yeh S., Wu C.-C., Miyamoto H., Merry D.E., Sobue G., Chen L.-M., Chang S.-S. (2007). ASC-J9 ameliorates spinal and bulbar muscular atrophy phenotype via degradation of androgen receptor. Nat. Med..

[32] Lee G.T., Nagaya N., Desantis J., Madura K., Sabaawy H.E., Kim W.-J., Vaz R.J., Cruciani G., Kim I.Y. (2020). Effects of MTX-23, a Novel PROTAC of Androgen Receptor Splice Variant-7 and Androgen Receptor, on CRPC Resistant to Second-Line Antiandrogen Therapy. Mol. Cancer Ther..

[33] Myung J.-K., Banuelos C.A., Fernandez J.G., Mawji N.R., Wang J., Tien A.H., Yang Y., Tavakoli I., Haile S., Watt K. (2013). An androgen receptor N-terminal domain antagonist for treating prostate cancer. J. Clin. Investig..

[34] Andersen R.J., Mawji N.R., Wang J., Wang G., Haile S., Myung J.-K., Watt K., Tam T., Yang Y.C., Bañuelos C.A. (2010). Regression of Castrate-Recurrent Prostate Cancer by a Small-Molecule Inhibitor of the Amino-Terminus Domain of the Androgen Receptor. Cancer Cell.

[35] Ravindranathan P., Lee T.-K., Yang L., Centenera M., Butler L., Tilley W., Hsieh J.-T., Ahn J.-M., Raj G.V. (2013). Peptidomimetic targeting of critical androgen receptor–coregulator interactions in prostate cancer. Nat. Commun..

[36] Chang C.-Y., McDonnell D.P. (2005). Androgen receptor–cofactor interactions as targets for new drug discovery. Trends Pharmacol. Sci..

[37] DePriest A.D., Fiandalo M., Schlanger S., Heemers F., Mohler J.L., Liu S., Heemers H.V. (2016). Regulators of Androgen Action Resource: A one-stop shop for the comprehensive study of androgen receptor action. Database.

[38] Gottlieb B., Beitel L.K., Nadarajah A., Paliouras M., Trifiro M. (2012). The androgen receptor gene mutations database: 2012 update. Hum. Mutat..

[39] Chmelar R., Buchanan G., Need E.F., Tilley W., Greenberg N.M. (2006). Androgen receptor coregulators and their involvement in the development and progression of prostate cancer. Int. J. Cancer.

[40] Liu S., Kumari S., Hu Q., Senapati D., Venkadakrishnan V.B., Wang D., DePriest A.D., Schlanger S.E., Ben-Salem S., Valenzuela M.M. (2017). A comprehensive analysis of coregulator recruitment, androgen receptor function and gene expression in prostate cancer. eLife.

[41] Ianculescu I., Wu D.Y., Siegmund K.D., Stallcup M.R. (2012). Selective roles for cAMP response element-binding protein binding protein and p300 protein as coregulators for androgen-regulated gene expression in advanced prostate cancer cells. J. Biol. Chem..

[42] Asangani I., Dommeti V.L., Wang X., Malik R., Cieslik M., Yang R., Escara-Wilke J., Wilder-Romans K., Dhanireddy S., Engelke C. (2014). Therapeutic targeting of BET bromodomain proteins in castration-resistant prostate cancer. Nature.

[43] Waddell A., Huang H., Liao D. (2021). CBP/p300: Critical Co-Activators for Nuclear Steroid Hormone Receptors and Emerging Therapeutic Targets in Prostate and Breast Cancers. Cancers.

[44] Link K.A., Balasubramaniam S., Sharma A., Comstock C., Godoy-Tundidor S., Powers N., Cao K.H., Haelens A., Claessens F., Revelo M.P. (2008). Targeting the BAF57 SWI/SNF Subunit in Prostate Cancer: A Novel Platform to Control Androgen Receptor Activity. Cancer Res..

[45] Fields S., Song O.-K. (1989). A novel genetic system to detect protein–protein interactions. Nature.

[46] Rao M.A., Cheng H., Quayle A.N., Nishitani H., Nelson C.C., Rennie P.S. (2002). RanBPM, a Nuclear Protein That Interacts with and Regulates Transcriptional Activity of Androgen Receptor and Glucocorticoid Receptor. J. Biol. Chem..

[47] He B., Bai S., Hnat A.T., Kalman R.I., Minges J.T., Patterson C., Wilson E.M. (2004). An Androgen Receptor NH2-terminal Conserved Motif Interacts with the COOH Terminus of the Hsp70-interacting Protein (CHIP). J. Biol. Chem..

[48] Wang X., Yeh S., Wu G., Hsu C.-L., Wang L., Chiang T., Yang Y., Guo Y., Chang C. (2001). Identification and Characterization of a Novel Androgen Receptor Coregulator ARA267-α in Prostate Cancer Cells. J. Biol. Chem..

[49] Sharma M., Zarnegar M., Li X., Lim B., Sun Z. (2000). Androgen Receptor Interacts with a Novel MYST Protein, HBO1. J. Biol. Chem..

[50] Grötsch H., Kunert M., Mooslehner K.A., Gao Z., Struve D., Hughes I.A., Hiort O., Werner R. (2012). RWDD1 interacts with the ligand binding domain of the androgen receptor and acts as a coactivator of androgen-dependent transactivation. Mol. Cell. Endocrinol..

[51] Obendorf M., Meyer R., Henning K., Mitev Y.A., Schröder J., Patchev V.K., Wolf S.S. (2007). FoxG1, a member of the forkhead family, is a corepressor of the androgen receptor. J. Steroid Biochem. Mol. Biol..

[52] Clark E.L., Coulson A., Dalgliesh C., Rajan P., Nicol S.M., Fleming S., Heer R., Gaughan L., Leung H.Y., Elliott D. (2008). The RNA Helicase p68 Is a Novel Androgen Receptor Coactivator Involved in Splicing and Is Overexpressed in Prostate Cancer. Cancer Res..

[53] Harada N., Takagi T., Nakano Y., Yamaji R., Inui H. (2015). Protein arginine methyltransferase 10 is required for androgen-dependent proliferation of LNCaP prostate cancer cells. Biosci. Biotechnol. Biochem..

[54] Wafa L.A., Cheng H., Rao M.A., Nelson C.C., Cox M., Hirst M., Sadowski I., Rennie P.S. (2003). Isolation and identification of l-dopa decarboxylase as a protein that binds to and enhances transcriptional activity of the androgen receptor using the repressed transactivator yeast two-hybrid system. Biochem. J..

[55] Hu B., Hu H., Yin M., Sun Z., Chen X., Li Y., Sun Z., Liu C., Li L., Qiu Y. (2018). Sertad1 promotes prostate cancer progression through binding androgen receptor ligand binding domain. Int. J. Cancer.

[56] Tavassoli P., Wafa L.A., Cheng H., Zoubeidi A., Fazli L., Gleave M., Snoek R., Rennie P.S. (2010). TAF1 Differentially Enhances Androgen Receptor Transcriptional Activity via Its N-Terminal Kinase and Ubiquitin-Activating and -Conjugating Domains. Mol. Endocrinol..

[57] Young K.H. (1998). Yeast two-hybrid: So many interactions, (in) so little time. Biol. Reprod..

[58] Stagljar I., Korostensky C., Johnsson N., Heesen S.T. (1998). A genetic system based on split-ubiquitin for the analysis of interactions between membrane proteins in vivo. Proc. Natl. Acad. Sci. USA.

[59] Ray M.R., Wafa L.A., Cheng H., Snoek R., Fazli L., Gleave M., Rennie P.S. (2006). Cyclin G-associated kinase: A novel androgen receptor-interacting transcriptional coactivator that is overexpressed in hormone refractory prostate cancer. Int. J. Cancer.

[60] Yeh S., Chang C. (1996). Cloning and characterization of a specific coactivator, ARA70, for the androgen receptor in human prostate cells. Proc. Natl. Acad. Sci. USA.

[61] Sundell G.N., Ivarsson Y. (2014). Interaction Analysis through Proteomic Phage Display. BioMed Res. Int..

[62] Sidhu S.S., Koide S. (2007). Phage display for engineering and analyzing protein interaction interfaces. Curr. Opin. Struct. Biol..

[63] Danner S., Belasco J.G. (2001). T7 phage display: A novel genetic selection system for cloning RNA-binding proteins from cDNA libraries. Proc. Natl. Acad. Sci. USA.

[64] Castillo J., Goodson B., Winter J. (2001). T7 displayed peptides as targets for selecting peptide specific scFvs from M13 scFv display libraries. J. Immunol. Methods.

[65] Müller J.M., Isele U., Metzger E., Rempel A., Moser M., Pscherer A., Breyer T., Holubarsch C., Buettner R., Schüle R. (2000). FHL2, a novel tissue-specific coactivator of the androgen receptor. EMBO J..

[66] Heery D., Kalkhoven E., Hoare S., Parker M.G. (1997). A signature motif in transcriptional co-activators mediates binding to nuclear receptors. Nature.

[67] Nolte R.T., Wisely G.B., Westin S., Cobb J.E., Lambert M.H., Kurokawa R., Rosenfeld M.G., Willson T.M., Glass C.K., Milburn M.V. (1998). Ligand binding and co-activator assembly of the peroxisome proliferator-activated receptor-gamma. Nature.

[68] Shiau A.K., Barstad D., Loria P.M., Cheng L., Kushner P.J., Agard D.A., Greene G.L. (1998). The Structural Basis of Estrogen Receptor/Coactivator Recognition and the Antagonism of This Interaction by Tamoxifen. Cell.

[69] He B., Kemppainen J.A., Wilson E.M. (2000). FXXLF and WXXLF Sequences Mediate the NH2-terminal Interaction with the Ligand Binding Domain of the Androgen Receptor. J. Biol. Chem..

[70] Dehm S.M., Regan K.M., Schmidt L.J., Tindall D.J. (2007). Selective Role of an NH2-Terminal WxxLF Motif for Aberrant Androgen Receptor Activation in Androgen Depletion–Independent Prostate Cancer Cells. Cancer Res..

[71] Hur E., Pfaff S.J., Payne E.S., Grøn H., Buehrer B., Fletterick R.J. (2004). Recognition and Accommodation at the Androgen Receptor Coactivator Binding Interface. PLoS Biol..

[72] Kazmin D., Prytkova T., Cook C.E., Wolfinger R., Chu T.-M., Beratan D., Norris J., Chang C.-Y., McDonnell D.P. (2006). Linking Ligand-Induced Alterations in Androgen Receptor Structure to Differential Gene Expression: A First Step in the Rational Design of Selective Androgen Receptor Modulators. Mol. Endocrinol..

[73] Chang C.-Y., Abdo J., Hartney T., McDonnell D.P. (2005). Development of Peptide Antagonists for the Androgen Receptor Using Combinatorial Peptide Phage Display. Mol. Endocrinol..

[74] Hsu C.-L., Liu J.-S., Wu P.-L., Guan H.-H., Chen Y.-L., Lin A.-C., Ting H.-J., Pang S.-T., Yeh S.-D., Ma W.-L. (2014). Identification of a new androgen receptor (AR) co-regulator BUD31 and related peptides to suppress wild-type and mutated AR-mediated prostate cancer growth via peptide screening and X-ray structure analysis. Mol. Oncol..

[75] Ozers M.S., Marks B.D., Gowda K., Kupcho K.R., Ervin K.M., De Rosier T., Qadir N., Eliason H.C., Riddle A.S.M., Shekhani M.S. (2007). The Androgen Receptor T877A Mutant Recruits LXXLL and FXXLF Peptides Differently than Wild-Type Androgen Receptor in a Time-Resolved Fluorescence Resonance Energy Transfer Assay. Biochemistry.

[76] Blessing A., Ganesan S., Rajapakshe K., Sung Y.Y., Bollu L., Shi Y., Cheung E., Coarfa C., Chang J.T., McDonnell D.P. (2015). Identification of a Novel Coregulator, SH3YL1, That Interacts with the Androgen Receptor N-Terminus. Mol. Endocrinol..

[77] Senapati D., Kumari S., Heemers H.V. (2020). Androgen receptor co-regulation in prostate cancer. Asian J. Urol..

[78] Norris J.D., Joseph J.D., Sherk A.B., Juzumiene D., Turnbull P.S., Rafferty S.W., Cui H., Anderson E., Fan D., Dye D.A. (2009). Differential Presentation of Protein Interaction Surfaces on the Androgen Receptor Defines the Pharmacological Actions of Bound Ligands. Chem. Biol..

[79] Groner A.C., Cato L., de Tribolet-Hardy J., Bernasocchi T., Janouskova H., Melchers D., Houtman R., Cato A.C., Tschopp P., Gu L. (2016). TRIM24 Is an Oncogenic Transcriptional Activator in Prostate Cancer. Cancer Cell.

[80] He H.-J., Gu X.-F., Xu W.-H., Yang D.-J., Wang X.-M., Su Y. (2013). Krüppel-like factor 8 is a novel androgen receptor co-activator in human prostate cancer. Acta Pharmacol. Sin..

[81] Mayeur G.L., Kung W.-J., Martinez A., Izumiya C., Chen D.J., Kung H.-J. (2005). Ku Is a Novel Transcriptional Recycling Coactivator of the Androgen Receptor in Prostate Cancer Cells. J. Biol. Chem..

[82] Chen S.-T., Okada M., Nakato R., Izumi K., Bando M., Shirahige K. (2015). The Deubiquitinating Enzyme USP7 Regulates Androgen Receptor Activity by Modulating Its Binding to Chromatin. J. Biol. Chem..

[83] Drake J.M., Graham N.A., Lee J.K., Stoyanova T., Faltermeier C.M., Sud S., Titz B., Huang J., Pienta K.J., Graeber T.G. (2013). Metastatic castration-resistant prostate cancer reveals intrapatient similarity and interpatient heterogeneity of therapeutic kinase targets. Proc. Natl. Acad. Sci. USA.

[84] Drake J., Graham N.A., Stoyanova T., Sedghi A., Goldstein A., Cai H., Smith D.A., Zhang H., Komisopoulou E., Huang J. (2012). Oncogene-specific activation of tyrosine kinase networks during prostate cancer progression. Proc. Natl. Acad. Sci. USA.

[85] Wang X.-L., Wen X.-F., Li R.-B., Liu B., Qiu G.-M., Wen J.-L., Wang Y.-M. (2014). Chrebp regulates the transcriptional activity of androgen receptor in prostate cancer. Tumor Biol..

[86] Mohammed H., D’Santos C., Serandour A.A., Ali R., Brown G.D., Atkins A., Palacio O.R., Holmes K.-A., Theodorou V., Robinson J.L. (2013). Endogenous purification reveals GREB1 as a key estrogen receptor regulatory factor. Cell Rep..

[87] Mohammed H., Taylor C., Brown G.D., Papachristou E., Carroll J., D’Santos C.S. (2016). Rapid immunoprecipitation mass spectrometry of endogenous proteins (RIME) for analysis of chromatin complexes. Nat. Protoc..

[88] Paltoglou S., Das R., Townley S.L., Hickey T., Tarulli G., Coutinho I., Fernandes R., Hanson A.R., Denis I., Carroll J. (2017). Novel Androgen Receptor Coregulator GRHL2 Exerts Both Oncogenic and Antimetastatic Functions in Prostate Cancer. Cancer Res..

[89] Augello M.A., Liu D., Deonarine L.D., Robinson B.D., Huang D., Stelloo S., Blattner M., Doane A.S., Wong E.W., Chen Y. (2019). CHD1 Loss Alters AR Binding at Lineage-Specific Enhancers and Modulates Distinct Transcriptional Programs to Drive Prostate Tumorigenesis. Cancer Cell..

[90] Branon T.C., Bosch J.A., Sanchez A.D., Udeshi N.D., Svinkina T., Carr S.A., Feldman J.L., Perrimon N., Ting A.Y. (2018). Efficient proximity labeling in living cells and organisms with TurboID. Nat. Biotechnol..

[91] Cho K.F., Branon T.C., Udeshi N.D., Myers S.A., Carr S.A., Ting A.Y. (2020). Proximity labeling in mammalian cells with TurboID and split-TurboID. Nat. Protoc..

[92] Vélot L., Lessard F., Bérubé-Simard F.-A., Tav C., Neveu B., Teyssier V., Boudaoud I., Dionne U., Lavoie N., Bilodeau S. (2021). Proximity-dependent Mapping of the Androgen Receptor Identifies Kruppel-like Factor 4 as a Functional Partner. Mol. Cell. Proteom..

[93] Hsiao J.J., Ng B.H., Smits M.M., Martinez H.D., Jasavala R.J., Hinkson I.V., Fermin D., Eng J., Nesvizhskii A.I., Wright M.E. (2015). Research Resource: Androgen Receptor Activity Is Regulated Through the Mobilization of Cell Surface Receptor Networks. Mol. Endocrinol..

[94] Geng C., He B., Xu L., Barbieri C., Eedunuri V.K., Chew S.A., Zimmermann M., Bond R., Shou J., Li C. (2013). Prostate cancer-associated mutations in speckle-type POZ protein (SPOP) regulate steroid receptor coactivator 3 protein turnover. Proc. Natl. Acad. Sci. USA.

[95] Mellacheruvu D., Wright Z., Couzens A.L., Lambert J.-P., St-Denis N.A., Li T., Miteva Y.V., Hauri S., Sardiu M.E., Low T.Y. (2013). The CRAPome: A contaminant repository for affinity purification–mass spectrometry data. Nat. Methods.

[96] Holding A.N. (2015). XL-MS: Protein cross-linking coupled with mass spectrometry. Methods.

[97] Dotzlaw H., Papaioannou M., Moehren U., Claessens F., Baniahmad A. (2003). Agonist–antagonist induced coactivator and corepressor interplay on the human androgen receptor. Mol. Cell. Endocrinol..

[98] Stelloo S., Nevedomskaya E., Kim Y., Hoekman L., Bleijerveld O.B., Mirza T., Wessels L.F.A., Van Weerden W.M., Altelaar A.F.M., Bergman A.M. (2018). Endogenous androgen receptor proteomic profiling reveals genomic subcomplex involved in prostate tumorigenesis. Oncogene.

[99] Stelloo S., Bergman A.M., Zwart W. (2019). Androgen receptor enhancer usage and the chromatin regulatory landscape in human prostate cancers. Endocr. Relat. Cancer.

[100] Fletterick R.J. (2005). Molecular modelling of the androgen receptor axis: Rational basis for androgen receptor intervention in androgen-independent prostate cancer. Br. J. Urol..

[101] Chang C.Y., McDonnell D.P. (2002). Evaluation of ligand-dependent changes in AR structure using peptide probes. Mol. Endocrinol..

[102] Yang W., Briegel A. (2018). Use of Cryo-EM to Study the Structure of Chemoreceptor Arrays In Vivo. Methods Mol. Biol..

[103] Iacobucci C., Piotrowski C., Aebersold R., Amaral B.C., Andrews P., Bernfur K., Borchers C., Brodie N.I., Bruce J.E., Cao Y. (2019). First Community-Wide, Comparative Cross-Linking Mass Spectrometry Study. Anal. Chem..

[104] Zheng J., Corzo C., Chang M.R., Shang J., Lam V.Q., Brust R., Blayo A.L., Bruning J.B., Kamenecka T.M., Kojetin D.J. (2018). Chemical Crosslinking Mass Spectrometry Reveals the Conformational Landscape of the Activation Helix of PPARgamma; a Model for Ligand-Dependent Antagonism. Structure.

[105] Wei L., Wang J., Lampert E., Schlanger S., DePriest A.D., Hu Q., Gomez E.C., Murakam M., Glenn S.T., Conroy J. (2017). Intratumoral and Intertumoral Genomic Heterogeneity of Multifocal Localized Prostate Cancer Impacts Molecular Classifications and Genomic Prognosticators. Eur. Urol..

[106] Malovannaya A., Lanz R.B., Jung S.Y., Bulynko Y., Le N.T., Chan D.W., Ding C., Shi Y., Yucer N., Krenciute G. (2011). Analysis of the Human Endogenous Coregulator Complexome. Cell.

